# The rising crisis of illicit fentanyl use, overdose, and potential therapeutic strategies

**DOI:** 10.1038/s41398-019-0625-0

**Published:** 2019-11-11

**Authors:** Ying Han, Wei Yan, Yongbo Zheng, Muhammad Zahid Khan, Kai Yuan, Lin Lu

**Affiliations:** 1 National Institute on Drug Dependence and Beijing Key Laboratory of Drug Dependence, Peking University, 100191 Beijing, China; 2 Peking University Sixth Hospital, Peking University Institute of Mental Health, NHC Key Laboratory of Mental Health (Peking University), National Clinical Research Center for Mental Disorders (Peking University Sixth Hospital), 100191 Beijing, China; 3 Peking-Tsinghua Center for Life Sciences and PKU-IDG/McGovern Institute for Brain Research, Peking University, 100871 Beijing, China

**Keywords:** Addiction, Pharmacodynamics

## Abstract

Fentanyl is a powerful opioid anesthetic and analgesic, the use of which has caused an increasing public health threat in the United States and elsewhere. Fentanyl was initially approved and used for the treatment of moderate to severe pain, especially cancer pain. However, recent years have seen a growing concern that fentanyl and its analogs are widely synthesized in laboratories and adulterated with illicit supplies of heroin, cocaine, methamphetamine, and counterfeit pills, contributing to the exponential growth in the number of drug-related overdose deaths. This review summarizes the recent epidemic and evolution of illicit fentanyl use, its pharmacological mechanisms and side effects, and the potential clinical management and prevention of fentanyl-related overdoses. Because social, economic, and health problems that are related to the use of fentanyl and its analogs are growing, there is an urgent need to implement large-scale safe and effective harm reduction strategies to prevent fentanyl-related overdoses.

## Introduction

Fentanyl was first developed in 1960 by Paul Janssen as a potent opioid anesthetic and analgesic. At the time, fentanyl was the fastest-acting opioid discovered to date and more powerful than morphine (50–100 times) and heroin (30–50 times)^1,2^. Transdermal, intravenous, and transbuccal fentanyl administration and several other drugs with chemical structures that are similar to fentanyl have been developed, approved, and used for surgical anesthesia and the management of severe cancer pain and perioperative pain, eventually becoming the most often used synthetic opioid in clinical practice^3–5^. Since 1979, fentanyl and its analogs have been synthesized in laboratories and sold as heroin substitutes or mixed with other illicitly sourced drugs, leading to an increase in fentanyl-related overdose deaths^6,7^. Postmortem studies have consistently found pulmonary edema, congestion, and needle puncture sites in these victims. Based on data from the National Vital Statistics System, 599,255 drug overdose deaths occurred from 1979 to 2016 in the United States, and the overall mortality rate has seen exponential growth. Fentanyl-related overdose deaths predominantly occurred in the northeastern United States, mostly affecting younger people (20–40 years of age), and grew sharply since 2013^8^.

Rapid death from ingesting fentanyl has become increasingly more common. Its high potency, fast onset of action, and duration of the desired effect may be particularly important contributing factors to the higher risk of overdose deaths and social consequences^9^. Fentanyl has become a major contributor to cocaine-related fatal overdoses. The rate of fentanyl-related overdose deaths increased 55% between 2015 and 2017 in New York city^10,11^. Synthetic opioids are also increasingly detected in illicit supplies of heroin, methamphetamine, and counterfeit pills. Analysis of a sampling of 1 million unique patients’ urine drug test (UDT) specimens showed that positivity rates for fentanyl have increased by 1850% among cocaine positive UDT results and increased by 798% among methamphetamine-positive UDT results between January 2013 and September 2018^12^. This mixture may lead to the increases in cocaine-related and methamphetamine-related overdoses. Moreover, the number of fatal overdoses from synthetic opioids, primarily fentanyl and its analogs, was 19,547 in 2016 in the United States, and this rate increased by 88% per year from 2013 to 2016^13–16^. The incidence of heroin-related overdose deaths stabilized in 2017, whereas deaths that involved other synthetic opioids continued to increase^17^. Given the substantial individual and public health threats of this emerging problem, the present review summarizes the epidemic and evolution of illicit fentanyl use, its pharmacological mechanism of action, its adverse consequences, and the clinical management and prevention of fentanyl-related overdoses.

## Epidemic and evolution of illicit fentanyl use

Fentanyl is currently approved and commonly used to treat breakthrough pain in cancer patients and various other clinical conditions that involve noncancer pain, such as postoperative pain. However, its potential for abuse and the rise in overdose deaths pose a serious challenge to public health^18–21^. Deaths that were attributable to illicit fentanyl use were first reported in the early 1980s and occurred sporadically in the United States^6,7,22^. A surge in the occurrence of fentanyl-related fatalities among illicit drug users occurred in 2006. A total of 1013 deaths in six states occurred from April 4, 2005, to March 28, 2007^23^. Since then, the prevalence of opioid-related mortality has increased persistently, and the number of reported fentanyl-related deaths more than doubled (from 2628 to 5544) between 2012 and 2014^21,24,25^. The rate of fentanyl-related overdose deaths increased from <15% in 2010 to ~50% in 2017 in Marion County, Indiana^26^. Overall overdose deaths and first-responder calls increased in a community-based sample in an impoverished neighborhood in Vancouver, Canada, in 2017, and fentanyl was detected in 52% of the subjects who were prescribed opioid agonist therapy^27^. At the same time, fentanyl-related deaths also increased in Australia^28,29^.

The presence of fentanyl and its analogs has become a central contributor to the increase in the number of opioid-related overdose deaths. Preliminary estimates of opioid overdose deaths in the United States in 2016 revealed that fentanyl and its analogs (e.g., acetylfentanyl, furanylfentanyl, and carfentanil) have contributed to nearly half of opioid overdose deaths^16,30,31^. Moreover, the number of deaths that were attributable to illicitly manufactured fentanyl and its analogs nearly quadrupled between July 2015 and June 2017 in Montgomery County, Ohio^32^. Heroin-positive cases declined while methamphetamine-positive cases increased in these victims. Urine drug screens showed that the prevalence of recent fentanyl use in patients who received opioid agonist treatment in England was 3%, and multiple fatalities with synthetic fentanyl analogs were reported in northern England in early 2017^33,34^.

Fentanyl is ~30–50 times more potent than heroin, and smaller volumes of heroin and other drugs that are adulterated with fentanyl can produce powerful effects with lower production costs. Detecting fentanyl and its analogs in used syringes can reveal exposure risk^35^. The fentanyl detection rate was significantly higher among drug users who injected drugs in the past 6 months compared with non-injection drug users. The prevalence of non-fatal overdose is very high among people who inject drugs^36–38^. The prevalence of intravenous fentanyl use among people who inject drugs in Australia is 8%. Given the narrow range between effective and lethal doses, this population is at high risk of overdose^37,39,40^. The opioid crisis is likely attributable to illicitly manufactured fentanyl and its analogs around the world, especially when they are mixed with heroin and other drugs, and the route of administration^41,42^.

Many people who have survived fentanyl overdose appear to be unaware that they ever took the drug. Surveys from 17 harm reduction sites in British Columbia, Canada, revealed that the prevalence of fentanyl use was 29% (70/242; based on urine drug screen), 73% of whom report that they did not knowingly use fentanyl^43^. Urine drug screens in methadone-maintained patients in Wayne County, Michigan, showed that 38% of 368 unique patients tested positive for fentanyl, and 67.3% of 113 patients reported that they did not know anyone who sought to obtain fentanyl in a subsequent anonymous survey^44^. A high risk of overdose and deaths was found among this vulnerable population that exhibited high fentanyl exposure, thus highlighting the pressing need to develop appropriate harm-reduction strategies, such as surveillance, the development of early-warning systems, pill-testing technology about the presence of fentanyl in various drug products, naloxone training and distribution, overdose education, and urine screens^21,45,46^. The vast majority of people reported their willingness to use rapid test strips to detect the presence of fentanyl in drugs or urine at home or utilize drug-checking services at supervised injection clinics^47,48^. Multiplex ultrahigh-performance liquid chromatography (UHPLC–MS)/liquid chromatography tandem mass spectrometry (LC–MS–MS)/liquid chromatography–quadrupole time-of-flight–mass spectrometry (LC–QTOF–MS) analyses have also been developed and validated for the detection of fentanyl and its analogs and metabolites in blood, hair, and oral fluid, which will be helpful for informing harm reduction behaviors and combating the fentanyl crisis^49–52^. A newly developed lateral flow immunoassay was also evaluated for effectiveness in the detection of fentanyl analogs^53^.

## Pharmacological mechanisms and side effects of fentanyl

Despite the beneficial clinical anesthetic and pain-relieving effects of fentanyl, the frequent use of fentanyl primarily affects the central nervous system (CNS) and gastrointestinal, cardiovascular, and pulmonary systems and can cause several side effects^54^. Digestive symptoms, such as nausea, vomiting, and constipation, are common in patients who repeatedly use fentanyl^55,56^. Immunosuppression was also shown to be precipitated by analgesic opioid drugs, including fentanyl, in preclinical and clinical studies. Such immunosuppression can be especially dangerous in the elderly and already immunocompromised patients^57–59^. Additionally, fentanyl and synthetic opioids have other frequently reported side effects, including migraine, dizziness, vertigo, confusion, hallucinations, and a higher risk of fractures in the elderly^59–63^. Fentanyl has rewarding effects and thus high abuse potential. Its repeated use leads to the development of tolerance and drug dependence^64,65^. Analyses of adverse-event reporting systems in the United States, Europe, and the United Kingdom have shown that cases of fentanyl-related misuse, abuse, dependence, and withdrawal steadily increased between 2004 and 2018, resulting in prolonged hospitalization or death^66^. Other mental disorders, such as depression, insomnia, and suicidality, can also occur with fentanyl abuse, contributing to relapse and a higher risk of respiratory depression or overdose death^65,67^. The treatment of these mental disorders may help prevent fentanyl-related fatalities and achieve abstinence.

Fentanyl is a full μ-opioid receptor agonist, but it also acts on δ- and κ-opioid receptors^68,69^. Fentanyl has been shown to exert its analgesic and lethal effects through different receptor populations in the CNS. It is eliminated from cerebrospinal fluid at approximately the same rate as morphine^70,71^. Acute naloxone administration antagonizes fentanyl-induced analgesia more than fentanyl-induced lethality. β-funaltrexamine was shown to inhibit both fentanyl-induced analgesia and lethality^71^. Overdose-related concentrations of fentanyl were shown to block human ether-a-go-go-related gene (hERG) potassium channels in ventricular myocytes that were isolated from neonatal rats, which may contribute to fentanyl-related overdose death or sudden death^72^.

Respiratory depression is the most dangerous adverse reaction to fentanyl that can result in lethality. In rats, intravenous injections of fentanyl dose-dependently decreased oxygen levels in the nucleus accumbens, basolateral amygdala, and subcutaneous space, followed by a delayed increase in glucose and fluctuations in brain temperature and metabolic brain activity^73–75^. Neuronal hypermetabolism that is induced by fentanyl and its analogs may damage the hippocampus and limbic system, causing an amnestic syndrome in patients who use fentanyl^76–79^. With regard to brain hypoxia and hypothermia, fentanyl has synergistic effects with heroin, which is consistent with the higher risk of overdose death that is associated with heroin–fentanyl mixtures^73,80^. Fentanyl-related respiratory depression is also dose-dependent, which reaches a peak 5 min after administration and requires 4 h to recover in humans. Such effects can lead to prolonged apnea and sudden death^74,81,82^. Epidural fentanyl infusion has been shown to cause postoperative adult respiratory distress syndrome^83^. The μ_1_-opioid receptor is involved in respiratory depression that is induced by fentanyl and its analogs but not morphine^84^. Selective α4β2 nicotinic receptor agonist A85380 reversed fentanyl-induced respiratory depression in rats without significant side effects^85^. The calcium-activated potassium channel blocker GAL021 was shown to attenuate morphine-induced respiratory depression in rats, mice, and nonhuman primates, and it produced stimulatory effects during alfentanil-induced respiratory depression, without affecting sedation in humans^86–88^. However, more studies are needed to confirm the efficacy and potential toxicity of A85380 and GAL021.

Many studies have reported cardiovascular symptoms after fentanyl-induced analgesia, such as myocardial ischemia, QTc interval prolongation, and bradycardia^89–91^. Fentanyl is commonly used during percutaneous coronary interventions, but the relative safety of its use requires further investigation because intravenous fentanyl has been reported to induce hypothermia, impair ticagrelor absorption, and cause antiplatelet effects^92–94^. Autopsy and toxicological analyses indicated that chronic fentanyl use may be responsible for hypertrophy, cardiac fibrosis, and atherosclerosis^54,95,96^. Neither sigma nor opioid receptors are essential for the fentanyl-induced attenuation of muscarinic coronary contraction^97^.

Fentanyl administration provides effective pain relief, but its long-term use can result in a lowering of pain thresholds^98,99^. This phenomenon of fentanyl-induced hyperalgesia is a challenge in the clinical management of perioperative and chronic pain. Recent studies showed that fentanyl-induced hyperalgesia was modulated by the activation of extracellular signal-regulated kinase in the laterocapsular division of the central nucleus of the amygdala (CeLC) and CaMKIIα in the CeLC–periaqueductal gray–rostral ventromedial medulla–spinal cord descending facilitative pain pathway in rats^100,101^.

## Interventions for the management and prevention of fentanyl overdose

Similar treatments are prescribed for opioid use disorder and opioid overdose, including the Food and Drug Administration (FDA)-approved medications methadone, buprenorphine, extended-release naltrexone, and naloxone^102^. Lofexidine, a central α_2_-adrenergic receptor agonist, was the first non-opioid medication that was approved by the United States FDA for the treatment of opioid withdrawal^103,104^. Lofexidine has fewer prescriptive barriers and comparable efficacy and safety relative to other opioid receptor agonizts, but it is generally more expensive. Sparse data are available on the effectiveness of interventions to prevent overdoses that are caused by illicitly manufactured fentanyl (Table 1). Compared with other opioid-related overdoses, illicit fentanyl-related overdoses appear to be accompanied by distinct symptoms, such as body and chest rigidity, dyskinesia, and slow or irregular heart rate, which can affect overdose management, such as oxygen provisions and appropriate doses of naloxone^105,106^. To avoid or reduce the adverse effects of fentanyl, the FDA proposed to control the duration of use and doses of fentanyl^107^. One study showed that the majority of patients who were presumed to experience fentanyl overdose could be discharged after brief emergency room observation, thus unlikely requiring additional naloxone dosing in the emergency room^108^.

**Table 1:** Overview of medications for the treatment of opioid use disorder and potential implications for the treatment of fentanyl overdose

Medication	Mechanism of action	Treatment	Limitations	Implications for fentanyl overdose
Methadone	Full MOR agonist	Reduces cravings and withdrawal symptoms.	Risk of dependence and acute withdrawal after abrupt discontinuation. Respiratory depression and QTc prolongation as a result of methadone overdose or illicit use.	Protects against death and promotes abstinence in fentanyl-exposed patients, but relapse rates are still high.
Buprenorphine	MOR partial agonistKOR antagonist	Reduces cravings and withdrawal symptoms.	Risk of acute withdrawal in OUD patients with high levels of tolerance.	Promotes treatment retention and opioid abstinence in fentanyl-exposed patients.
Extended-release naltrexone	MOR antagonistKOR antagonist	Reduces cravings, promotes abstinence, promotes treatment retention, and prevents relapse.	Requires detoxification before initiating naltrexone treatment.High risk of early induction failure.	Adverse events, including overdoses, did not differ between extended-release naltrexone and buprenorphine-naloxone combination.
Naloxone	MOR antagonist	Reduces craving, promotes abstinence, promotes treatment retention, and prevents relapse.	Risk of precipitating opioid withdrawal.	Mostly utilized to reverse the overdose epidemic, but its efficacy needs improvement, and safe dosing needs further investigation.
Lofexidine	Central α_2_-adrenergic receptor agonist	Reduces withdrawal symptoms but not drug craving.	Hypotension and bradycardia. Not effective for all withdrawal symptoms.	N/A

*MOR* μ-opioid receptor, *KOR* κ-opioid receptor, *OU*D opioid use disorder

There are limited data on the efficacy of methadone or buprenorphine for the treatment of illicit fentanyl use. A retrospective study in Rhode Island showed that 6 months of methadone maintenance protected against death and promoted abstinence in fentanyl-exposed patients, but relapse rates were still high^109^. Buprenorphine is a μ-opioid receptor partial agonist and κ-opioid receptor antagonist that is commonly used to treat opioid use disorder. It also exerts antidepressant and anxiolytic activity and is a promising treatment for neonatal opioid withdrawal syndrome^110^. A retrospective cohort study showed that 6-month treatment retention rates and opioid abstinence rates were not different between individuals who were positive for fentanyl or heroin at baseline before initiating buprenorphine treatment, indicating that buprenorphine may still be beneficial for treating fentanyl exposure^111^. Repeated treatment with buprenorphine produced a greater magnitude of antinociceptive tolerance than higher-efficacy agonizts (e.g., morphine and etonitazene) in rats^112^. Studies in pigeons and rhesus monkeys showed that the amount of tolerance that develops to the reinforcing potency of opioids depends on their efficacy, and the higher-efficacy μ-opioid receptor agonist sufentanil was more difficult to antagonize than the low-efficacy μ-opioid receptor agonist morphine^113–115^. These data indicate that buprenorphine may have lower efficacy for the treatment of fentanyl overdose compared with heroin overdose, although no human trials have been performed to date^116^.

Naloxone is a μ-opioid receptor antagonist that is used to treat fentanyl-related overdose, regardless of the suspected route of administration. However, its efficacy is inconsistent, and safe dosing needs to be considered from the perspective of precipitating opioid withdrawal^117–119^. Recent studies also showed that extended-release naltrexone was equally safe and effective as a buprenorphine–naloxone combination at promoting abstinence and treatment retention once treatment was initiated, but fewer participants successfully initiated naltrexone treatment^120,121^. Larger or repeated doses of naloxone are speculated to be required for the treatment of fentanyl overdose because of its higher affinity for μ-opioid receptors. However, a study of a community naloxone distribution program in Allegheny County showed that the average doses of naloxone that were administered to reverse overdose did not change between 2013 and 2016, although the incidence of overdoses that were related to fentanyl and its analogs increased during the same time^122^. A retrospective study of the fentanyl epidemic in Chicago showed that doses of naloxone up to 12 mg may effectively treat fentanyl overdose^123^. Naloxone was shown to reverse transdermal fentanyl overdose-induced sedation, the reduction of body temperature, and the reduction of heart rate in dogs^124^. A systematic review found a low incidence of mortality or serious adverse events that were caused by prehospital naloxone administration in opioid overdose patients, although the source of overdose was mostly heroin and not fentanyl^125^. Additionally, seeking emergency medical help was positively associated with overdose victims who received higher doses of naloxone and rescue breathing in British Columbia, Canada^126^. A survey of 316 street-recruited people who used opioids in Baltimore showed that the majority of them perceived the high risk of fentanyl-adulterated heroin and overdose, but most of them did not often carry naloxone with them^127^. The early adoption and distribution of take-home naloxone have been reported to effectively prevent opioid overdose deaths^128–130^. Therefore, harm reduction strategies, such as safe injection sites, the expansion of available opioid agonist treatment, and overdose prevention training (e.g., carrying naloxone and not use drugs alone, higher dose or multiple administrations of naloxone), are needed to control the adverse effects of fentanyl and reduce overdoses^131^.

Additionally, more potent, longer-acting opioid receptor antagonists are needed to prevent fentanyl-related overdose deaths. Compared with naloxone, nalmefene has been shown to have superior efficacy in reversing the carfentanil-induced loss of righting reflex and respiratory depression in rats^132^. Nalmefene is generally well tolerated and is a recent option for patients with alcohol dependence^133–135^. Additionally, novel, selective, and potent μ-opioid receptor antagonists, such as 17-cyclopropylmethyl-3,14β-dihydroxy-4,5α-epoxy-6α-(isoquinoline-3-carboxamido)morphinan (NAQ) and 17-cyclopropylmethyl-3,14β-dihydroxy-4,5α-epoxy-6α-(indole-7-carboxamido)morphinan (NAN), have been reported to produce less opioid tolerance, dependence, and withdrawal signs. Furthermore, NAN pretreatment was shown to block the discriminative stimulus effects of fentanyl in rats. The orexin-1 receptor antagonist SB-334867 was also shown to decrease motivation and demand for fentanyl in rats^136^. Therefore, these drugs could be considered candidates for the treatment of opioid use disorder^137^. Chronic anticonvulsant carbamazepine therapy was shown to increase fentanyl clearance and decrease plasma concentrations in neurosurgical patients, which may attenuate the actions of fentanyl^138^. A case report showed that treatment with slow-release oral morphine in a near-fatal fentanyl overdose patient was successful, despite the patient’s previous failures with methadone and buprenorphine/naloxone-based opioid agonist therapies, which could be considered potential alternative treatments^139^.

Previous studies have reported the vaccine consisting of fentanyl hapten conjugated to tetanus toxoid or keyhole limpet hemocyanin carrier protein, and immunization with these vaccines reduced fentanyl biodistribution to the brain, and blunted its antinociceptive effects and respiratory depression in rodents^140,141^. Moreover, the conjugate vaccine stimulated the endogenous generation of antibodies with high affinity for a variety of fentanyl analogs^140^, and was shown to blunt fentanyl reinforcement^142^. A recent study screened and purified monoclonal antibodies (mAbs) from vaccinated mice, and found that the 6A4 mAb prevented the acute lethality of fentanyl, and reversed both fentanyl and carfentanil-induced antinociception as effective as naloxone^143^. These findings suggest that immunopharmacotherapies including active vaccine or its combination with passive mAb may be potential and promising treatment strategies to address the current opioid crisis. Accumulating evidence also implicate the dysbiosis of gut microbiome in the pathophysiology of drug addiction, however data regarding fentanyl use is rare^144^. Manipulating the compositions of the gut microbiome or its products may guide new adjuvant therapies for opioid addiction in the future.

A United States FDA Risk Evaluation and Mitigation Strategy (REMS) program was also implemented to assess transmucosal immediate-release fentanyls (TIRFs) and found that substantial rates of TIRFs were prescribed inappropriately^145,146^. With the findings of deficiencies in the structure and administration of TIRFs, the development of other REMSs is needed to ensure the safe and appropriate use of approved drugs, especially dangerous opioid drugs^147^.

In conclusion, the crisis of opioid-related overdoses, especially fentanyl and its analogs, is a major threat to both individual and public health. Respiratory depression, cardiovascular effects, and neuropsychiatric symptoms are associated with fentanyl overdose and lethality. Naloxone is the standard rescue drug for fentanyl overdose, but its efficacy is inconsistent. Further clinical research is needed to optimize individualized medication-assisted treatments in patients who overdose on fentanyl and its analogs. To address the social, economic, and health problems that are associated with fentanyl and its analogs, coordinated efforts are needed to implement large-scale harm reduction strategies (e.g., naloxone distribution, innovative studies, and the development of novel drugs).
